# Bicycle-related hospitalizations at a Taiwanese level I Trauma Center

**DOI:** 10.1186/s12889-015-2075-9

**Published:** 2015-07-29

**Authors:** Hang-Tsung Liu, Cheng-Shyuan Rau, Chi-Cheng Liang, Shao-Chun Wu, Shiun-Yuan Hsu, Hsiao-Yun Hsieh, Ching-Hua Hsieh

**Affiliations:** Department of Trauma Surgery, Kaohsiung Chang Gung Memorial Hospital and Chang Gung University College of Medicine, No.123, Ta-Pei Road, Niao-Song District, Kaohsiung City, 833 Taiwan; Department of Neurosurgery, Kaohsiung Chang Gung Memorial Hospital and Chang Gung University College of Medicine, Kaohsiung City, Taiwan; Department of Anesthesiology, Kaohsiung Chang Gung Memorial Hospital and Chang Gung University College of Medicine, Kaohsiung City, Taiwan

**Keywords:** Bicyclist, Motorcyclist, Helmet, Head injury, Injury severity score, Mortality, Trauma registry system

## Abstract

**Background:**

This study aimed to investigate differences in injury severity and mortality between patients who met with bicycle or motorcycle accidents and were hospitalized at a Level I trauma center in Taiwan.

**Methods:**

We performed a retrospective analysis of bicycle-related injuries that have been reported in the Trauma Registry System in order to identify and compare 699 bicyclists to 7,300 motorcyclists who were hospitalized for treatment between January 1, 2009 and December 31, 2013. Statistical analyses of the injury severity, associated complications, and length of stay in the hospital and intensive care unit (ICU) were performed to compare the risk of injury of bicyclists to that of motorcyclists with the corresponding unadjusted odds ratios and 95 % confidence intervals (CIs). Adjusted odds ratios (AORs) and 95 % CIs for mortality were calculated by controlling for confounding variables that included age, and an Injury Severity Score (ISS) was calculated.

**Results:**

More of the cyclists were under 19 years of age or over 70 than were the motorcyclists. In contrast, fewer bicyclists than motorcyclists wore helmets, arrived at the emergency department between 11 p.m. and 7 a.m., and had a positive blood alcohol concentration test. The bicyclists sustained significantly higher rates of injuries to the extremities, while motorcyclists sustained significantly higher rates of injuries to the head and neck, face, and thorax. Compared to motorcyclists, the bicyclists had significantly lower ISSs and New Injury Severity Scores, shorter length hospital stays, and a smaller proportion of admittance into the ICU. However, the bicyclists had higher AORs for in-hospital mortality (AOR: 1.2, 95 % CI: 1.16–1.20). In terms of critical injury severity (ISS ≥ 25), the bicyclists had 4.4 times (95 % CI: 1.95–9.82) the odds of mortality than motorcyclists with the same ISSs.

**Conclusions:**

Data analysis indicated that the bicyclists had unique injury characteristics including bodily injury patterns and lower ISSs, but had higher in-hospital mortality compared to motorcycle riders. In this study, given that only 9 % of bicyclists reported wearing helmets and considering the high mortality associated with head injury, it is possible that some bicycle riders underestimated the gravity of cycling accidents.

## Background

Bicycles are a popular form of transport and recreation worldwide. However, cyclists are likely to suffer more severe consequences of road accidents than users of motorized vehicles. Road accidents involving cyclists have widely varying consequences, ranging from minor abrasions to fractures and death. One nationally representative study found that bicyclists had 2.3 times as many fatalities and 1.8 times as many nonfatal injuries as motor vehicle occupants per 100 million person-trips [1]. It is estimated that for every two million trips, 600 injuries will occur and one bicyclist will die in a crash [2]. Approximately a third of the injured cyclists required hospitalization [3, 4]. In the United States, there were more than 25,000 bicycling-related hospitalizations annually between 2002 and 2009 [5]. These hospitalizations accounted for a national estimate of one billion dollars in total hospital charges per year [5].

To improve the safety of cyclists, multiple governing bodies have been involved in planning bicycle-friendly urban spaces, designing traffic management solutions at intersections and bicycle roundabouts, and maintaining cycle paths [6]. The investment in infrastructure development significantly improved the safety of cyclists and lead to a steady decrease in the number of bicycle fatalities and road fatalities in general [6]. However, because of the growing popularity of bicycle transportation and the rising number of cyclists, a high incidence of bicycle-related injuries is still observed [2]. The identification of high-risk injury patterns and a greater understanding of major trauma epidemiology are vital in order to maximize the provision of services and the quality of care delivered [7, 8]. In addition, compared to the many studies that have focused on accident and injury rates, relatively few studies have focused on cyclist injury severity. It was previously reported that the median Injury Severity Score (ISS) was four (range 1–41) for individuals with bicycle-related injuries who required hospitalization in the United Arab Emirates [9]. At one university hospital in Japan, the average ISS was 23.9 for 115 bicyclists who died as a consequence of traffic accidents [10]. When speeding was involved in bicycle-motor vehicle collisions, the probability of a fatal injury increased by 300 % [11]. Given that most bicycle accidents occur on relatively crowded streets in Asian cities, bicycle-related injuries occur at relatively low velocities, similar to most motorcycle-related injuries, which represent a major proportion of traffic accident-related hospital admissions in South Taiwan [12]. Therefore, the aim of this study was to investigate differences in injury severity and mortality between patients who sustained bicycle or motorcycle accidents and were hospitalized at a Level I trauma center in Taiwan using data from a population-based trauma registry.

## Methods

### Ethics statement

This study was pre-approved by the Institutional Review Board (IRB) of the hospital (approval number 103-4599B). Informed consent was waived according to IRB regulations.

### Study design

#### Data

In this retrospective study, all data added to the Trauma Registry System of a 2,400-bed Level I regional trauma center, which provided care to trauma patients who were primarily from South Taiwan, were reviewed. Cases involving hospitalization for trauma sustained in motorcycle accidents between January 1, 2009 and December 31, 2013 were selected. Among the 16,548 hospitalized and registered patients who were entered into the database, 669 (4.0 %) were bicyclists (which included 657 bicyclists and 12 moped riders and accounted for the legal speed limit of < 25 km/h in Taiwan) and 7,300 (44.1 %) were motorcyclists (which included motorcycle, motorized tricycle, and all-terrain vehicle riders).

#### Study variables

Detailed patient information was obtained from our institutional Trauma Registry System, which included patient age, sex, arrival time, mode of transportation, vital signs upon admission, collision manner, and helmet use. A blood alcohol concentration (BAC) of 50 mg/dL, the legal limit for drivers in Taiwan, was defined as the threshold. Other data collected included the first emergency department Glasgow Coma Scale (GCS) assessment, details of the emergency procedures performed (i.e. cardiopulmonary resuscitation, intubation, chest tube insertion, and blood transfusion), an Abbreviated Injury Scale (AIS) for each body region, the Injury Severity Score (ISS), New Injury Severity Score (NISS), Trauma Injury Severity Score (TRISS), length of hospital stay (LOS), LOS in the intensive care unit (ICU), in-hospital mortality, and associated complications. Stratified ISS data were compared to identify differences in injury severity using clinically relevant ISS cutoffs: ≥ 16 for severe and ≥ 25 for critical injuries. In our study, the primary outcome was injury severity as measured by various scoring systems (GCS, AIS, ISS, NISS, and TRISS) and in-hospital mortality. The secondary outcomes were the associated complications, and hospital and ICU LOS.

#### Exploratory analysis

The data collected were analyzed using the SPSS v.20 statistical software (IBM, Armonk, NY). Chi-square tests were used to determine the significance of associations between the predictor and outcome variables among the categorical variables. Student t-tests were used evaluate the significance of associations between the predictor and outcome variables among the continuous variables. Univariate logistic regression analyses were initially performed to identify the significant predictor variables of the injury or mortality risk of bicyclists. The corresponding unadjusted odds ratios (ORs) with 95 % confidence intervals (CIs) for each variable were obtained.

#### Regression analysis

The adjusted odds ratios (AORs) and 95 % CIs for mortality were estimated through stepwise model selection of a multiple regression model that was adjusted by controlling the cofounding variables of age and ISS. All of the results are presented as the mean ± standard error. A p-value < 0.05 was considered statistically significant.

## Results

### Patient characteristics

As shown in Table 1, the mean patient ages were 50.7 ± 24.8 and 42.3 ± 19.0 years in the bicyclist and motorcyclist groups, respectively. Among the bicyclists, a greater number of patients were aged 0–9, 10–19, 70–79, 80–89, and ≥90 years, and fewer patients were aged 20–29, 30–39, and 40–49 years. More of the bicyclists than motorcyclists were children, teenagers, or elderly. No statistically significant difference regarding sex was identified between bicyclists and motorcyclists. The helmet-wearing status was recorded as 99.6 % and 97.7 % for bicyclists and motorcyclists, respectively; however, at the time of injury, significantly fewer bicyclists were wearing helmets than motorcyclists (9.0 % vs. 84.7 %, respectively; p < 0.001). Most of the bicyclists and motorcyclists arrived at the emergency department between 7 a.m. and 5 p.m., while more of the motorcyclists arrived between 11 p.m. and 7 a.m. (p < 0.001). In comparison to motorcyclists, more injured bicyclists were transported to the hospital in a private vehicle (OR: 2.2, 95 % CI: 1.85–2.64; p < 0.001) and less injured bicyclists were transported by emergency medical services (OR: 0.6, 95 % CI: 0.48–0.67; p < 0.001). More bicycle accidents occurred as a result of riders losing control (including sliding and turn-over), while more motorcycle accidents involved collisions with cars, buses, trucks, or obstacles (such as a wall, tree, pillar, or pedestrian). A positive BAC test result was less frequent among bicyclists than among motorcyclists (4.2 % vs. 9.6 %, respectively; p < 0.001).

**Table 1:** Demographics of bicyclists hospitalized due to trauma

Variables	Bicycle	Motor	*Odds ratio*	*p*
	*n* = 669	*n* = 7300	*(95 % CI)*	
Age	50.7±24.8	42.3±19.0	-	<0.001
Age category				
0-9years	40(6.0)	75(1.0)	6.1(4.14-9.07)	<0.001
10-19years	106(15.8)	823(11.3)	1.5(1.19-1.85)	<0.001
20-29 years	16(2.4)	1575(21.6)	0.1(0.05-0.15)	<0.001
30-39 years	32(4.8)	947(13.0)	0.3(0.24-0.48)	<0.001
40-49 years	65(9.7)	1015(13.9)	0.7(0.51-0.87)	0.002
50-59 years	108(16.1)	1268(17.4)	0.9(0.74-1.14)	0.422
60-69 years	107(16.0)	980(13.4)	1.2(0.99-1.53)	0.064
70-79yeas	135(20.2)	503(6.9)	3.4(2.77-4.21)	<0.001
80-89years	56(8.4)	106(1.5)	6.2(4.44-8.66)	<0.001
≧90 years	4(0.6)	8(0.1)	5.5(1.65-18.26)	0.002
Gender				0.141
Male	410(61.3)	4260(58.4)	1.1(0.96-1.33)	--
Female	259(38.7)	3040(41.6)	0.9(0.75-1.04)	--
Helmet				
Yes	60(9.0)	6185(84.7)	0.02(0.01-0.02)	<0.001
No	606(90.6)	946(13.0)	64.6(49.41-84.49)	<0.001
Unknown	3(0.4)	169(2.3)	0.2(0.06-0.60)	0.001
Time				
7:00–17:00	194(29.0)	2145(29.4)	1.0(0.82-1.17)	0.834
17:00–23:00	383(57.2)	3909(53.5)	1.2(0.99-1.36)	0.066
23:00–7:00	92(13.8)	1239(17.0)	0.8(0.62-0.98)	0.033
Unknown	0(0.0)	7(0.1)	-	0.423
Transportation				
Private vehicle	202(30.2)	1195(16.4)	2.2(1.85-2.64)	<0.001
EMS	235(35.1)	3575(49.0)	0.6(0.48-0.67)	<0.001
Transferred	232(34.7)	2530(34.7)	1.0(0.85-1.18)	0.991
Mechanism, hit with				
Bicycle	11(1.6)	75(1.0)	1.6(0.85-3.05)	0.139
Motorcycle	144(21.5)	1663(22.8)	0.9(0.77-1.13)	0.458
Car	89(13.3)	2346(32.1)	0.3(0.26-0.41)	<0.001
Bus or Truck	11(1.6)	372(5.1)	0.3(0.17-0.57)	<0.001
Out of control	387(57.8)	2105(28.8)	3.4(2.88-3.98)	<0.001
Obstacle	27(4.0)	739(10.1)	0.4(0.25-0.55)	<0.001
BAC > 50 mg/dL, n(%)	28(4.2)	700(9.6)	0.4(0.28-0.61)	<0.001

We found a significant difference in the GCS between bicyclists and motorcyclists (14.3 ± 2.2 vs. 14.2 ± 2.5, respectively; *p* = 0.045) (Table 2), as well as in the distribution of scores among patients with a GCS ≥ 13. However, the difference in GCS between bicyclists and motorcyclists was less than one point. In contrast, there was no difference in the proportion of patients with a GCS of either ≤ 8 or 9–12 between bicyclists and motorcyclists. Our analysis of the AIS scores revealed that bicyclists sustained significantly higher rates of injury to the extremities, while motorcyclists sustained significantly higher rates of injury to the head or neck, face, and thorax. The comparison of injury scores between the bicyclists and motorcyclists indicated significant differences in the ISS (8.7 ± 7.1 vs. 9.6 ± 7.7, respectively; p < 0.001). When stratified by injury severity (ISS < 16, 16–24, or ≥ 25), more bicyclists had an ISS of less than 16 compared to motorcyclists (86.7 % vs. 82.0 %, respectively; *p* = 0.002), while more motorcyclists had an ISS of 16–24, compared to bicyclists (12.5 % vs. 9.4 %, respectively; *p* = 0.021). There was no significant difference in these two groups of patients with an ISS of ≥ 25. We also found significant differences between bicyclists and motorcyclists regarding the NISS (10.1 ± 9.1 vs. 11.2 ± 9.3, respectively; *p* < 0.001), TRISS (0.949 ± 0.137 vs. 0.960 ± 0.112, respectively; *p* = 0.033), and in-hospital mortality rates (2.8 % vs. 1.7 %, respectively; *p* = 0.030). Among patients with critical injuries (ISS ≥ 25), bicyclists had a higher OR (OR: 4.4, 95 % CI: 1.95–9.82; p < 0.001) of mortality than motorcyclists. However, no difference was found between the injured cyclists and motorcyclists with ISSs of < 16 and from 16 to 24. After adjusting for age and ISS, we found that bicyclists had a significantly higher AOR for patient mortality than did motorcyclists (AOR: 1.2, 95 % CI: 1.16–1.20; p < 0.001), indicating that the differences in injury severity between bicyclists and motorcyclists were not entirely responsible for their distinct mortality rates.

**Table 2:** Injury severity and mortality of bicyclists hospitalized due to trauma

Variables	Bicycle	Motor	*Odds ratio*	*p*
	*n* = 669	*n* = 7300	*(95 % CI)*	
GCS	14.3±2.2	14.2±2.5	-	0.045
n (%)				
≤ 8	30(4.5)	435(6.0)	0.7(0.51-1.08)	0.119
9-12	23(3.4)	324(4.4)	0.8(0.50-1.18)	0.225
≥ 13	616(92.1)	6541(89.6)	1.3(1.01-1.80)	0.043
AIS, n (%)				
Head/Neck	189(28.3)	2411(33.0)	0.8(0.67-0.95)	0.012
Face	96(14.3)	1834(25.1)	0.5(0.40-0.62)	<0.001
Thorax	60(9.0)	1212(16.6)	0.5(0.38-0.65)	<0.001
Abdomen	31(4.6)	577(7.9)	0.6(0.39-0.82)	0.002
Extremity	492(73.5)	5274(72.2)	1.1(0.89-1.28)	0.473
ISS	8.7±7.1	9.6±7.7	-	<0.001
>16	580(86.7)	5986(82.0)	1.4(1.14-1.80)	0.002
16-24	63(9.4)	910(12.5)	0.7(0.56-0.96)	0.021
≧25	26(3.9)	404(5.5)	0.7(0.46-1.03)	0.071
NISS	10.1±9.1	11.2±9.3	-	<0.001
TRISS	0.949±0.137	0.960±0.112	-	0.033
Mortality, n (%)	19(2.8)	123(1.7)	1.7(1.05-2.78)	0.031
>16, n	2	9	2.3(0.50-10.67)	0.274
16-24, n	3	29	1.5(0.45-5.13)	0.498
≧25, n	14	85	4.4(1.95-9.82)	<0.001
AOR			1.2(1.16-1.20)	<0.001

The findings regarding the types of injuries associated with bicycle accidents are shown in Table 3. Bicyclists had a higher OR for sustained humeral fracture (OR: 2.2, 95 % CI: 1.65–2.80; p < 0.001) as well as radial (OR: 1.4, 95 % CI: 1.15–1.83; *p* = 0.002), ulnar (OR: 1.7, 95 % CI: 1.25–2.25; *p* = 0.001), and femoral fractures (OR: 1.6, 95 % CI: 1.32–2.02; *p* < 0.001). However, compared to motorcyclists, bicyclists had a significantly lower percentage of cranial, orbital, maxillary, mandibular, rib, clavicle, metacarpal, pelvic, tibial, and metatarsal fractures, as well as epidural hemorrhage, subarachnoid hemorrhage (SAH), hemothorax, hepatic injury, and splenic injury. A significantly shorter hospital LOS was found for bicyclists compared to motorcyclists (7.8 days vs. 9.8 days, respectively; *p* = 0.001) (Table 4). Moreover, a significantly smaller proportion of bicyclists than motorcyclists was admitted to the ICU (15.7 % vs. 19.1 %, respectively; *p* = 0.033). No differences were noted in the proportion of bicyclists and motorcyclists who were admitted into the ICU, or in the LOS in the ICU after the stratification into either group based on injury severity (ISS of < 16, 16–24, or ≥ 25).

**Table 3:** Associated injuries of bicyclists hospitalized due to trauma

Variables	Bicycle	Motor	*Odds ratio*	*p*
	*n* = 669	*n* = 7300	*(95 % CI)*	
Head/Neck trauma, n(%)				
Neurologic deficit	3(0.4)	66(0.9)	0.5(0.16-1.58)	0.223
Cranial fracture	36(5.4)	616(8.4)	0.6(0.44-0.87)	0.006
Epidural hematoma (EDH)	22(3.3)	382(5.2)	0.6(0.40-0.95)	0.028
Subdural hematoma (SDH)	65(9.7)	763(10.5)	0.9(0.71-1.20)	0.550
Subarachnoid hemorrhage (SAH)	63(9.4)	885(12.1)	0.8(0.58-0.99)	0.039
Intracerebral hematoma (ICH)	16(2.4)	182(2.5)	1.0(0.57-1.61)	0.872
Cerebral contusion	42(6.3)	441(6.0)	1.0(0.75-1.45)	0.806
Cervical vertebral fracture	9(1.3)	65(0.9)	1.5(0.75-3.06)	0.240
Maxillofacial trauma, n(%)				
Orbital fracture	6(0.9)	206(2.8)	0.3(0.14-0.70)	0.003
Maxillary fracture	29(4.3)	715(9.8)	0.4(0.29-0.61)	<0.001
Mandibular fracture	12(1.8)	269(3.7)	0.5(0.27-0.86)	0.011
Nasal fracture	5(0.7)	110(1.5)	0.5(0.20-1.21)	0.115
Rib fracture	41(6.1)	863(11.8)	0.5(0.35-0.67)	<0.001
Sternal fracture	0(0.0)	15(0.2)	-	0.241
Hemothorax	7(1.0)	159(2.2)	0.5(0.22-1.01)	0.050
Pneumothorax	8(1.2)	166(2.3)	0.5(0.26-1.06)	0.068
Hemopneumothorax	9(1.3)	129(1.8)	0.8(0.38-1.50)	0.423
Lung contusion	6(0.9)	119(1.6)	0.5(0.24-1.25)	0.144
Thoracic vertebral fracture	2(0.3)	54(0.7)	0.4(0.10-1.65)	0.191
Abdominal trauma, n(%)				
Intra-abdominal injury	7(1.0)	134(1.8)	0.6(0.26-1.21)	0.138
Hepatic injury	9(1.3)	216(3.0)	0.4(0.23-0.88)	0.016
Splenic injury	3(0.4)	119(1.6)	0.3(0.09-0.86)	0.017
Retroperitoneal injury	1(0.1)	15(0.2)	0.7(0.10-5.51)	0.757
Renal injury	4(0.6)	52(0.7)	0.8(0.30-2.33)	0.735
Urinary bladder injury	0(0.0)	18(0.2)	-	0.199
Lumbar vertebral fracture	5(0.7)	86(1.2)	0.6(0.26-1.56)	0.316
Sacral vertebral fracture	1(0.1)	45(0.6)	0.2(0.03-1.75)	0.127
Scrotum injury	0(0.0)	15(0.2)	-	0.241
Extremity trauma, n(%)				
Scapular fracture	12(1.8)	173(2.4)	0.8(0.42-1.36)	0.344
Clavicle fracture	50(7.5)	945(12.9)	0.5(0.40-0.73)	<0.001
Humeral fracture	73(10.9)	393(5.4)	2.2(1.65-2.80)	<0.001
Radial fracture	94(14.1)	740(10.1)	1.4(1.15-1.83)	0.002
Ulnar fracture	55(8.2)	370(5.1)	1.7(1.25-2.25)	0.001
Metacarpal fracture	7(1.0)	266(3.6)	0.3(0.13-0.60)	<0.001
Pelvic fracture	10(1.5)	275(3.8)	0.4(0.21-0.73)	0.002
Femoral fracture	113(16.9)	809(11.1)	1.6(1.32-2.02)	<0.001
Patella fracture	14(2.1)	207(2.8)	0.7(0.42-1.27)	0.263
Tibia fracture	44(6.6)	778(10.7)	0.6(0.43-0.81)	0.001
Fibular fracture	27(4.0)	420(5.8)	0.7(0.46-1.03)	0.065
Calcaneal fracture	29(4.3)	396(5.4)	0.8(0.54-1.16)	0.230
Metatarsal fracture	7(1.0)	201(2.8)	0.4(0.18-0.80)	0.008

**Table 4:** Length of stay (LOS) in the hospital and intensive care unit (ICU) of bicyclists hospitalized due to trauma

Variables	ISS	Bicycle	Motor	*Odds ratio*	*p*
		*n* = 669	*n* = 7300	*(95 % CI)*	
Hospital LOS		7.8±9.3	9.8±10.4	-	<0.001
ICU LOS		105(15.7)	1391(19.1)	0.8(0.64-0.98)	0.033
n (%)	<16	40(6.9)	472(7.9)	0.9(0.62-1.21)	0.397
	16-24	42(66.7)	574(63.1)	1.2(0.68-2.01)	0.568
	≥25	23(88.5)	345(85.4)	1.3(0.38-4.51)	0.666
days		7.2±8.8	7.3±8.8	-	0.907
	<16	4.9±5.0	5.2±8.8	-	0.406
	16-24	7.3±7.7	6.8±5.9	-	0.244
	≥25	11.2±13.2	10.9±6..8	-	0.942

The major injuries associated with mortality are listed in Table 5, and the data revealed that the bicyclists were significantly older (50.7 ± 24.8 and 42.3 ± 19.0 years, respectively; *p* = 0.001) and were more likely to ride without a helmet (89.5 % vs. 26.8 %, respectively; *p* = 0.001). There was no significant difference between the bicyclists and motorcyclists in terms of the collision mechanisms of accidents responsible for mortality. Of the 19 bicyclists and 123 motorcyclists who died, the bicyclists did not have higher odds for major injuries than the motorcyclists; however, they did have greater odds for sustaining SAH (OR: 2.8, 95 % CI: 1.00–7.76; *p* = 0.046).

**Table 5:** Demographics and associated injuries of bicycle-related fatalities

Variables	Bicycle	Motor	*Odds ratio*	*p*
	*n* = 19	*n* = 123	*(95 % CI)*	
Age	67.4±13.7	51.2±19.0	-	0.001
Gender				0.294
Male	11(57.9)	86(69.9)	0.6(0.22-1.59)	
Female	8(42.1)	37(30.1)	1.7(0.63-4.54)	
Helmet				
Yes	2(10.5)	62(50.4)	0.1(0.03-0.52)	0.001
No	17(89.5)	33(26.8)	23.2(5.08-105.83)	<0.001
Unknown	0(0.0)	28(22.8)	-	0.020
Mechanism				
Bicycle	0(0.0)	1(0.8)	-	0.693
Motorcycle	4(21.1)	17(13.8)	1.7(0.49-5.61)	0.409
Car	6(31.6)	42(34.1)	0.9(0.32-2.51)	0.826
Bus or Truck	2(10.5)	12(9.8)	1.1(0.22-5.29)	0.917
Out of control	3(15.8)	29(23.6)	0.6(0.17-2.23)	0.450
Others	4(21.1)	22(17.9)	1.2(0.37-4.05)	0.740
Head/Neck trauma, n(%)				
Cranial fracture	9(47.4)	40(32.5)	1.9(0.70-4.96)	0.205
Epidural hematoma (EDH)	7(36.8)	33(26.8)	1.6(0.58-4.39)	0.367
Subdural hematoma (SDH)	12(63.2)	67(54.5)	1.4(0.53-3.89)	0.478
Subarachnoid hemorrhage (SAH)	13(68.4)	54(43.9)	2.8(1.00-7.76)	0.046
Intracerebral hematoma (ICH)	4(21.1)	15(12.2)	1.9(0.56-6.56)	0.291
Cerebral contusion	8(42.1)	29(23.6)	2.4(0.87-6.42)	0.087
Cervical vertebral fracture	0(0.0)	5(4.1)	-	0.371
Thoracic trauma, n(%)				
Rib fracture	0(0.0)	14(11.4)	-	0.121
Sternal fracture	0(0.0)	1(0.8)	-	0.693
Hemothorax	0(0.0)	11(8.9)	-	0.175
Pneumothorax	0(0.0)	8(6.5)	-	0.252
Hemopneumothorax	0(0.0)	9(7.3)	-	0.223
Lung contusion	0(0.0)	16(13.0)	-	0.095
Thoracic vertebral fracture	0(0.0)	1(0.8)	-	0.693
Abdominal trauma, n(%)				
Intra-abdominal injury	2(10.5)	8(6.5)	1.7(0.33-8.64)	0.524
Hepatic injury	1(5.3)	15(12.2)	0.4(0.05-3.22)	0.374
Splenic injury	0(0.0)	8(6.5)	-	0.252
Retroperitoneal injury	0(0.0)	3(2.4)	-	0.491
Renal injury	0(0.0)	3(2.4)	-	0.491
Extremity trauma, n(%)				
Pelvic fracture	2(10.5)	8(6.5)	1.7(0.33-8.64)	0.524
Femoral fracture	2(10.5)	13(10.6)	1.0(0.21-4.80)	0.995

## Discussion

### Age distribution and injury region of the bicyclists

It was previously reported that older bicyclists were at a significantly higher risk for sustaining fractures to the upper extremities [13]. In addition, thoracic and abdominal injuries were rare among younger cyclists, except for in cases of isolated ruptures of the spleen or liver by bicycle handlebars [14]. In a study of 12,429 hospital admissions that resulted from bicycle-related injuries involving motor vehicles, three out of every 100 patients presented with a splenic injury, and two out of every 100 patients sustained a liver injury [13]. The analysis of AIS scores in this study revealed that bicyclists sustained significantly higher rates of injuries to the extremities, while motorcyclists sustained significantly higher rates of injuries to the head or neck, face, and thorax. Bicyclists had a higher OR for sustained humeral fracture (OR: 2.2, 95 % CI: 1.65–2.80), radial fracture (OR: 1.4, 95 % CI: 1.15–1.83), ulnar fracture (OR: 1.7, 95 % CI: 1.25–2.25), and femoral fracture (OR: 1.6, 95 % CI: 1.32–2.02) than motorcyclists. In contrast, the ORs of hepatic injury (OR: 0.4, 95 % CI: 0.23–0.88) and splenic injury (OR: 0.3, 95 % CI: 0.09–0.86) were significantly lower for bicyclists than for motorcyclists. In the present study, more cyclists were under 19 years of age or over 70 compared to motorcyclists. Cyclists who were greater than 60 years of age accounted for 45.2 % of all injured cyclists. In contrast, 21.9 % of all injured motorcyclists were > 60 years of age. In Taiwan, the rate of hip fracture is among the highest in the world, and the age-specific incidence rate of hip fracture has been found to increase exponentially with age for both sexes, after the age of 65 [15]. The larger number of elderly patients may, in part, explain the higher odds of extremity fractures for injured cyclists than that for motorcyclists.

### The incidence pattern of bicycle accidents

Prior studies have shown that motor vehicle collisions with cyclists have resulted in an increase in the overall severity of injury [16] and a 4.6-fold increase in the odds of serious injury [17] compared to non-collisions. Heavy vehicles such as trucks (to a large extent) and buses (to a lesser extent) have been associated with higher cyclist injury severity [17]. A recent study of trauma hospitalizations revealed that bicycle crashes involving motor vehicles resulted in a 10-fold greater risk of death in hospital for adults (95 % CI: 1.8–34.3) and a eight-fold greater risk for children under 17 years of age (95 % CI: 1.2–85.3) [18]. In this study, loss of control was the main cause of bicycle injuries and accounted for 57.8 % of all patients with bicycle-related injuries. Collision with a motorcycle (21.5 %) was the second most common cause of injury. While 1.6 % of accidents involved buses or trucks, an additional 13.3 % of accidents involved cars. The incidence of bicycle accidents involving motor vehicle collisions was markedly lower than in prior reports, potentially reflecting a distinct epidemiology of bicycle accidents in a relatively crowded city and resulting in bicyclists who presented with differences in injury severity.

### Head injury of the bicyclists

The main cause of death and moderate disability after bicycle-related incidents was head injury [19, 20]. The overall incidence of head injury was 28.3 %, starting at 29.9 % in the pediatric group and increasing to 38.6 % in the elderly population [13]. Furthermore, the nature of the intracranial injuries differed significantly between the various age groups. Although the incidence of epidural hematomas was similar across age strata, the incidence of other intracranial injuries such as subdural hematoma and SAH was found to increase proportionally with age [13]. In this study, head or neck injury was noted in 189 of 669 (28.3 %) bicyclists who were admitted to the hospital, a result that is comparable to a study in which approximately a third of the 1,859 patients who were hospitalized with bicycle-related injured had one or more head injuries [3]. In this study, the bicyclists who suffered fatal injuries were significantly older and neglected to wear a helmet than those motorcyclists. Notably, among the fatal cases, although bicyclists had a significantly lower percentage of SAH than motorcyclists, they had greater odds for sustaining SAH.

### Helmet use and the mortality of the bicyclists

Among the various preventive measures, wearing a helmet in particular has been shown to protect against head injuries in both groups of riders [21, 22]. The odds of sustaining a head injury increased 1.98–3.89 times for cyclists who did not wear a helmet [3]. In addition, compared to cyclists who did not wear helmets, helmeted cyclists were less likely to sustain serious bodily injuries other than to the head, less likely to disobey a traffic light, less likely to have a BAC over 0.05 mg/dL, and more likely to be riding during the day [3]. A case-controlled study demonstrated that wearing a helmet reduced the risk of head injury by 63 % and the risk of loss of consciousness by 86 % among children [23]. Moreover, the average number of deaths per year decreased by 52 % after the institution of a mandatory helmet law [24]. The present study revealed a very low rate (9 %) of helmet use among South Taiwanese cyclists in comparison to cyclists in Germany (12 %–15 %) [25, 26], Finland (13 %) [4], Canada (50 %) [27], the United States (54 %) [28], and the state of Victoria in Australia (75.4 %) [3], which in 1990 became one of the first regions worldwide to introduce mandatory helmet legislation for cyclists on public roadways. In Taiwan, helmet use is not mandatory and is only required for competitive cyclists. Although the Taiwanese Government has invested in infrastructure and has conducted health programs to promote bicycle safety, there are no compulsory helmet laws for cyclists, contrary to the laws for motorcyclists. Indeed, it is possible that some bicycle riders underestimated the seriousness of cycling accidents. In this study, although bicyclists had significantly lower ISSs than motorcyclists, their mortality rate was higher. In addition, bicyclists with critical ISSs (ISS ≥ 25) had approximately four times the odds of mortality than motorcyclists with ISSs. Without adequate protection, riding a bicycle is more dangerous than riding a motorcycle if you are severely injured.

### Limitations of the study

The limitations of this study include the retrospective design and the lack of available data regarding injury mechanisms and circumstances, including speed, helmet material, exposure data (e.g., number of trips, hours of riding, and/or miles traveled), and details regarding the accident location (e.g., infrastructure characteristics and land use, light and weather conditions, as well cyclist behavior and maneuvers). Additionally, the number of patients in the study was not adequate to analyze the association of age with different accident characteristics other than mortality. The relatively small number of hospitalized bicyclists precluded an in-depth examination of risk factors such as age, type of head injury, and injury severity. Finally, the injured patients who were pronounced dead at the scene of the accident or those who were discharged from the emergency department were not included in the sample, which may have introduced a survival bias.

## Conclusions

This study indicates that bicyclists have unique injury characteristics including bodily injury patterns, as well as lower ISSs but higher in-hospital mortality when compared to motorcycle riders. In the study, because only 9 % of bicyclists reported wearing a helmet, and considering the high mortality associated with head injury, it is possible that some bicycle riders underestimated the gravity of cycling accidents.
